# Implementation of a Savvy Mobile ECG Sensor for Heart Rhythm Disorder Screening at the Primary Healthcare Level: An Observational Prospective Study

**DOI:** 10.3390/mi12010055

**Published:** 2021-01-05

**Authors:** Staša Vodička, Antonija Poplas Susič, Erika Zelko

**Affiliations:** 1 Healthcare Center Murska Sobota, 9000 Murska Sobota, Slovenia; 2 Healthcare Center Ljubljana, 1000 Ljubljana, Slovenia; antonija.poplas-susic@zd-lj.si; 3 Department of Family Medicine, Medical Faculty, University of Maribor, 2000 Maribor, Slovenia; erika.zelko@um.si

**Keywords:** heart rhythm disorders, palpitations, primary healthcare, personal mobile ECG sensor, referrals

## Abstract

Introduction: The Jozef Stefan Institute developed a personal portable electrocardiogram (ECG) sensor Savvy that works with a smartphone, and this was used in our study. This study aimed to analyze the usefulness of telecardiology at the primary healthcare level using an ECG personal sensor. Methods: We included 400 patients with a history of suspected rhythm disturbance who visited their family physician at the Healthcare Center Ljubljana and Healthcare Center Murska Sobota from October 2016 to January 2018. Results: The study found that there was no statistically significant difference between the test and control groups in the number of present rhythm disorders and actions taken to treat patients with either observation or administration of a new drug. However, in the test group, there were significantly fewer patients being referred to a cardiologist than in the control group (*p* < 0.001). Discussion: The use of an ECG sensor helps family physicians to distinguish between patients who need to be referred to a cardiologist and those who can be treated by them. This method is useful for both physicians and patients because it shortens the time taken to start treatment, can be used during pandemics such as COVID-19, and reduces unnecessary cost.

## 1. Introduction

A heart rhythm disorder or arrhythmia is a condition involving a translation through the conduction system; therefore, it is not a normal sinus rhythm that begins with a pulse in the sinoatrial node in the upper right atrium [1,2,3,4,5]. Heart rhythm disorders affect millions of people annually. Approximately 50% of deaths in Europe are caused by cardiovascular diseases [6]. Some arrhythmias are extremely difficult to diagnose because they occur sporadically, and some may cause immediate danger to the patient’s health [7].

To diagnose heart rhythm disorder in a patient, the gold standard after taking a patient’s history and conducting a physical examination is to perform an electrocardiogram (ECG) using a standard 12-lead ECG recorder. One single record does not necessarily reflect the rhythm disturbance, which can be transient, so more continuous methods, such as Holter monitoring, are used, which can be a 24 or 48 h continuous measurement of the ECG signal [8]. To access Holter monitoring and additional diagnostic tools, patients are referred to cardiologists (at the secondary or tertiary healthcare level or in a hospital setting), which can create a flood of unnecessary referrals and cause longer waiting lists. 

Among the EU countries, Slovenia, with a total of two million inhabitants, has the lowest number of people who cannot afford healthcare services for financial reasons despite the good public healthcare system [9,10]. To maintain the quality of our public healthcare system, we need to act responsibly. To treat certain conditions at the primary healthcare level without unnecessary referral of patients to other healthcare levels, we need useful and affordable diagnostic tools. This has also proven especially useful during the COVID-19 pandemic, where measures are being taken to prevent the spread of this infectious disease using partial or full lockdown [11].

Various body sensors have been developed over recent years. They are small portable devices that can be used for measuring different bodily functions in vivo and can be placed on the body surface or implanted in the body surgically [12]. By measuring electrical signals on the skin, they can provide us with a range of information [13]. In the case of measuring heart rate, valuable information can be obtained about the health state of a person [14]. These sensors can be used to monitor healthy individuals during sport and fitness activities or to monitor patients with heart rhythm disorders. By combining a low-cost sensor with a smartphone, we can monitor heart rate effectively and affordably [15].

In collaboration with medical experts, the Jožef Stefan Institute of Slovenia developed a personal portable ECG sensor that works in conjunction with a smartphone [16]. The Personal Digital Mobile Body Sensor is owned by Savvy Slovenia. It includes a personal sensor, ECG electrode kit, MobECG mobile application, and computer program called VisECG, which is currently available for Android only [17]. The ECG sensor consists of two electrodes, which are placed 8.5 cm apart, and measures the potential between these two closely placed body-surface electrodes. The gadget weighs 21 g and has several possible positions of placement. The measurement is transmitted via Bluetooth connection to a smartphone, where the device is paired and managed via the MobECG mobile application. 

When reading the recording, one should remember that it is not any standard lead typically used in a Holter monitor and that it is a single-channel measurement that is performed live on a patient moving and performing daily activities. VisECG enables us to review and accurately analyze the recording and produce a report that can be submitted to physicians of other specialties for consultation or delivered to the patient and his or her physician as a measurement result. Given that the measurement is only a single channel, we can estimate the frequency and possible disturbances of rhythm but not ischemia.

The use of remote sensing and wireless technology for continuous monitoring of a patient’s vital signs and heart rhythm monitoring in hospital settings has already been proven very useful in shortening the time from diagnosis of acute myocardial infarction to necessary treatment [18], in reducing the effect of the patient’s distance from the hospital with the Cath Lab [19], in reducing mortality of patients with acute myocardial infarction [20], in screening patients with the intent to detect incident atrial fibrillation [21], and in the detection of arrhythmias in patients with palpitations and syncope [22,23,24]. Studies have also been conducted in the field of patient satisfaction [25,26] and cost-effectiveness [27]. However, there is a lack of well-planned interventional studies using telemedical and wearable devices at the primary level. Therefore, this study aimed to analyze the usefulness of using a telemedical device in primary healthcare settings (in the office of family physicians at the healthcare center), mainly how it affects the number of patients referred to a cardiologist. With the help of a questionnaire, we also aimed to evaluate the satisfaction of patients and family physicians with this new method. 

## 2. Participants and Methods

### 2.1. Study Design

This observational prospective randomized cohort study was conducted on patients with a history of rhythm disturbance who visited their family physician at the Healthcare Center of Ljubljana and Healthcare Center of Murska Sobota from October 2016 to January 2018. 

The study included the patients and about 30 of their family physicians currently working at the two healthcare facilities. Patients without previously known heart conditions were invited to join the study. All patients included in the study had complained of a heart rhythm disorder but had no detectable rhythm disturbance in the 12-channel ECG record after examination at the first visit. If a rhythm disturbance was detected at that time, the patient was treated according to the guidelines and was not included in the study. However, if a diagnosis of rhythm disorder could not have been made, the patient was included in the study. The inclusion criteria were age ≥18 years, suspected heart rhythm disorder, and no previous diagnosis of heart rhythm disorder. The exclusion criteria were age ≤18 years, known heart rhythm disorder or previous diagnosis of heart rhythm disorder, and cognitive and psychiatric disorders.

We divided participants into two groups, a test group where they received the personal ECG sensor and a control group without the sensor, one after another according to their visits to the office. The randomization strategy was that we included the first patient who visited the doctor in the test group, the second patient in the control group, then the third patient in the test group, the fourth in the control group, and so on. Each participant in the test group received an ECG sensor, which was placed by a healthcare professional and worn for 3 days, at which time they received information about the placement and handling of the sensor. The sensor was placed using two self-adhesive electrodes on the skin of the chest, ensuring that it was not too hairy. Then, the sensor was attached to the electrode and connected via Bluetooth to the smartphone using an application called MobECG, which was also provided to each patient with a charger for the duration of the examination. Patients were advised to perform their daily activities as regularly as possible and not to remove the sensor while showering or sleeping and to keep the smartphone in the same room as them. In case the electrode peeled off, the patient had four spare electrodes and was instructed on how to install them. During the 3-day sensor monitoring, each patient kept a journal wherein he or she wrote down his or her problems and feelings. After 3 days, the patient returned to the healthcare provider, who removed the sensor and downloaded the measurements from the phone, which were then sent to the physicians who analyzed the reading. Then, the measurement was transferred to the computer memory using a program called VisECG and sent to the physicians for evaluation by mail. Each patient then underwent a checkup with the physician after 5–10 days, depending on the date they had made an appointment. The physician read the recording, and in the case of severe rhythm disorder, the patient was called earlier for a checkup at the physician’s clinic where he or she received treatment as needed. Otherwise, the regular checkup after sensor removal was made 5 to 10 days later, depending on appointment scheduled at the time of removal. The control group received a questionnaire and returned to the physician after 5–10 days.

For the study, we used a validated questionnaire that was made for a similar pilot study in a hospital setting. On the first visit to the doctor’s office, all patients received a questionnaire to record their problems and provide an opinion on the quality and usefulness of the examination. The second questionnaire we used in the study was made for physicians, and they had to complete two questionnaires: one for a patient on the first visit after 5–10 days, and then a second one after 3 months on their gender, age, specialty, and years of service.

The questionnaire used provided us with demographics data of physicians and patients, patients’ chronic illness and medication, presence of possible rhythm disorder, and actions taken after the first and second visit. We also asked both physicians and patients to record their comments on the quality and usefulness of this method. With the help of Raosoft Sample Size Calculator, we estimated that a sample size of 400 patients (200 in the test and 200 in the control group) is sufficient to obtain relevant data in our population (sample size corresponds to a confidence level of more than 95% and 4.9% margin of error).

Patients who met our criteria were invited to participate in our study, which was approved by the Ethics Committee of the Republic of Slovenia (number 0120-299/2017-7, KME 47/06/17). Each patient received and signed a written informed consent form, confirming their participation. The study was conducted in accordance with The Code of Ethics of the World Medical Association (Declaration of Helsinki), and it was registered on ClinicalTrials.gov (ID: NCT04463524).

### 2.2. Analysis

The collected data were analyzed using IBM SPSS Statistics 26 (Statistical Package for the Social Sciences, SPSS^®^ Inc., Chicago, IL, USA).

In the descriptive analysis, in the case of normal data distribution, we used central tendency and variability, arithmetic mean, standard deviation, and minimum and maximum as numerical variables; in the case of asymmetric data distribution, the median and minimum and maximum were used; and for descriptive variables, we used frequencies and percentages.

The following tests were used in the univariant analysis:

- *t*-test of independent samples or ANOVA for normally distributed numerical variables;
- Mann–Whitney U-test for abnormally distributed numerical variables;
- Pearson chi-squared test for descriptive variables or Fisher’s exact test as a correction for smaller samples.

A *p*-value ≤ 0.05 was considered statistically significant in all tests.

## 3. Results

### 3.1. Participants

**Figure 1:**
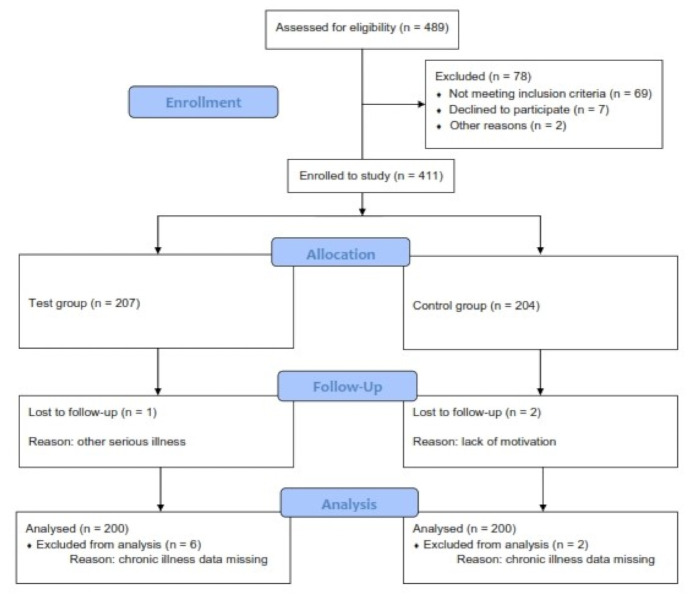
Study flow diagram.

As shown in the study flow diagram (Figure 1), we invited 498 patients to participate. 69 did not meet the inclusion criteria, 7 declined, and 2 were declined due to cognitive impairment and the inability to maintain follow up needed. In the study, we divided 411 participants into two groups: 207 in the test group and 204 in the control group; 3 were lost in the follow-up process because of lack of motivation and other serious illness. A total of 408 patients and their 32 family physicians were enrolled in the analysis, but complete data were obtained from only 400 patients and their 30 family physicians, which were included in our analysis. Data on patients’ chronic illness or therapy were missing for eight patients, and two physician questionnaires were not submitted.

### 3.2. Descriptive Data

Baseline characteristics for physicians and their patients are shown in Table 1, and those for patients are shown in Table 2.

Physicians involved were mainly family medicine specialists with 17.9 ± 11.17 years of working experience; there was no difference in gender and were aged from 27 to 61 years old.

The mean ± SD age of patients was 49.79 ± 16.36 years, and 101 out of 400 were male. There is no significant difference between the groups, but a higher percentage of women than men were involved in our study.

Out of 400 patients, 161 had finished university or had a higher education level, and 190 were working, 61 were unemployed, and others were retired or still in school. Moreover, 120 were receiving treatment for hypertension (mean blood pressure ± SD is 133.93 ± 16.33/82.99 ± 11.87 mmHg) and 54 for diabetes, 133 were smokers, and 250 were excessive drinkers (more than 1 unit of alcohol per day for a woman and more than 2 units of alcohol per day for a man, if 1 unit of alcohol equals 10 milliliters or 8 grams of pure alcohol).

There is only one significant difference between the groups, and that is the number of smokers, which is higher in the control group.

Heart Rhythm Detection Using a Personal Digital ECG Sensor

Table 3 shows the difference in the suspicion of rhythm disorder between the test and control groups; the proportion of patients with anamnestic suspicion in the test group was significantly lower compared to the control group (45.0% vs. 66.0%). The opposite is true for clinical suspicion, where the proportion of patients with this suspicion in the test group was significantly higher (more than double) compared to the control (34.0% vs. 16.0%).

The results showed that there were statistically significant differences in the type of suspicion between the sexes (according to the group; total: *p* < 0.001, men compared to women: *p* < 0.001). Men in the test group compared with men in the control group recorded a significantly higher proportion of clinical suspicion (40.0% vs. 27.0%) and both suspicions (19.0% vs. 0.0%) and a significantly lower proportion of anamnestic suspicion (40.0% vs. 73.0%). Clear differences (otherwise slightly different from men) were found for women. Compared to the control group, they recorded a significantly higher share of clinical suspicion (31.0% vs. 12.0%) and a lower share of anamnestic suspicion (47.0% vs. 64.0%) and both suspicions (20.0% vs. 23.0%), where the difference is almost negligible.

After the ECG sensor measurements and the first checkups at a family physician, we found no differences confirmed as statistically significant for the presence of rhythm disturbances between the two groups (Table 4). Secondly, the results show that there were statistically significant differences in the type of action between the groups primarily from referrals to a cardiologist. The test group recorded a significantly lower proportion of patients referred to cardiologists compared to the control group (11.5% vs. 32.0%). Differences in type of action between sexes were confirmed as significant (total: *p* < 0.001, male: *p* = 0.009, women: *p* < 0.001).

Men in the test group recorded a significantly higher proportion of having had a new drug treatment prescribed (9.0% vs. 4.0%) and being only observed (16.0% vs. 8.0%) but a lower proportion of patients referred to a cardiologist (3.5% vs. 10.0%). For women, slight differences were observed: women in the test group recorded a significantly lower proportion of patients referred to a cardiologist (3.5% vs. 22.0%) and being only observed (39.0% vs. 54.0%) but a higher proportion of patients given new drug treatments (18.5% vs. 0.0%).

### 3.3. Questionnaires

**Figure 2:**
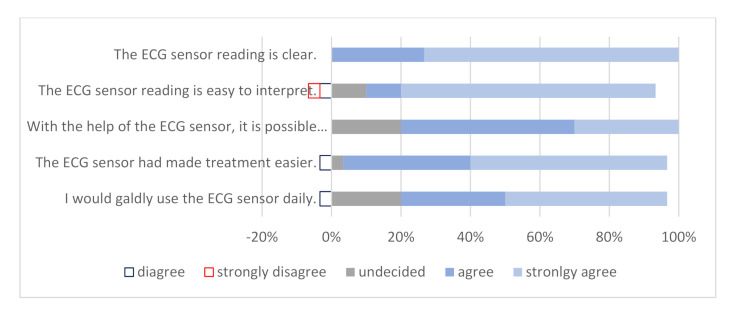
Physician satisfaction with ECG sensor use.

**Figure 3:**
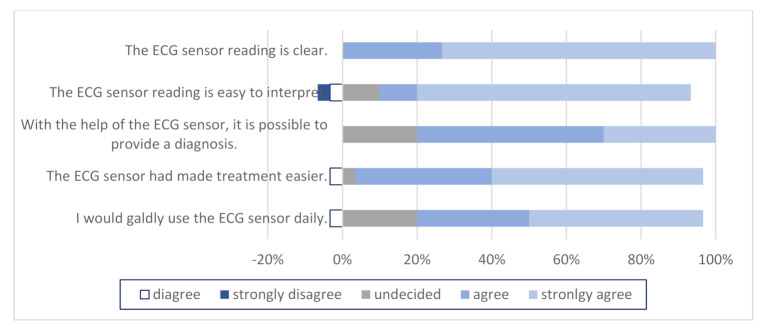
Patient satisfaction with ECG sensor use.

A questionnaire using a Likert scale was also included in our study about the satisfaction with this new method of using an ECG sensor to detect heart rhythm disorder. Results were collected from physicians (Figure 2) and patients (Figure 3). Most physicians agree that the ECG sensor reading is clear and easy to interpret, can help them to provide an accurate diagnosis, and made patient treatment easier, and they would gladly use ECG sensor daily. Almost all patients revealed that the ECG sensor was easy to install and use, they were not bothered by the ECG sensor and had no problems in using it, and they gladly wore the ECG sensor.

## 4. Discussion

The major finding in our study was in the number of referrals to cardiologists. There was a statistically significant difference in the number of patients being referred to a cardiologist (*p* < 0.001) between the test and control groups. In the test group, physicians referred 11.5% of patients, but in the control group, the number increased to 32% of patients. In order for this to be of any importance, we had to prove that this new method of using an ECG sensor is as good as the standard actions taken (12-lead ECG record, referral to cardiologist). After ECG sensor measurement and the first checkup at a family physician, we found that there was no statistically significant difference in the number of patients with present rhythm disorders between the test and control groups; therefore, we proved that this is a good and reliable screening method for heart rhythm disorders in the primary healthcare system. This makes the patient and physician satisfaction concerning the use of a personal ECG sensor of even greater importance. In our study, 82–98% of patients revealed that the use of an ECG sensor was easy, they were not bothered by the ECG sensor, and they gladly wore it and had no problems in its use.

Many studies have been conducted in this field in a hospital setting, but there are few well-structured studies at the primary healthcare level. Approximately 50% of deaths in Europe are caused by cardiovascular diseases [6]. Our study found that only 26–32% of patients with a complaint of heart rhythm disorders have a rhythm disturbance like those in previous studies, such as the one conducted in Germany, where patients also had to have a sinus rhythm at the time of ECG measurement, after which a patient-activated event recorder was used [20]. The study concluded that 61.4% of patients did not have heart rhythm disorders, 14.7% had atrial fibrillation, and 6.5% had ventricular rhythm disorder. Our conclusion is similar to the following findings [20]: there was a significant difference between the test and control groups, although 74% of patients in the test group and 68% of patients in the control group did not have a heart rhythm disorder; 6–8% had a ventricular rhythm disorder; and 12% had atrial fibrillation. Patients with complaints of palpitations also represent a significant burden to the healthcare system, as palpitations are one of the most common reasons why family physicians refer patients to the secondary level for cardiologist examination.

The treatment in our study is similar to that of the abovementioned study: physicians changed the patient’s pharmacotherapy in 88.7% of cases, and 26.8% of patients were referred to a psychiatrist or psychotherapist. In our case, physicians treated their patients the same as in the test and control groups; they changed their therapy or continued monitoring in 62–74% of patients, and 2–10% were referred to a specialist other than a cardiologist.

The major difference in our study was in the number of referrals to cardiologists. There was a statistically significant difference in the number of patients being referred to a cardiologist in the test and control groups. Concerning the lack of the blinding of the physician choosing which intervention to follow, we must report that this was done only to ensure the patients’ safety. If a patient complained about discomfort in their chest, and the physician established that it was an atypical form of chest pain, then the physician had the opportunity to assign this patient to the control group rather than sending the patient to sensor placement. Studies have shown that patients presenting with atypical chest pain are more likely to be suffering from an anxiety disorder, and when the standard 12-lead ECG reading is normal, they do not have a greater risk for cardiovascular incidents [28,29].

We noticed that there were some statistical differences between the test and control groups. Patients involved in the control group were older, their blood pressure was higher, and more of them were smokers. After considering that patients were mainly in the age group from 47 to 51 years of age, we can rule out the effect of this statistically significant difference because there is no clinical difference in this age group [30]. In both groups, the level of alcohol consumption was high. According to our National Institute of Public Health, out of every 10 inhabitants of Slovenia, two never drink alcohol, seven consume alcohol at a harmful level, and one is addicted to alcohol [31]. There were some differences between the sexes, but the number of male participants was low, and it was not a representative sample. Since the study was randomized and included patients who visited their doctor at that time, we could not control the number of male patients being involved, and studies have shown that patients presenting with palpitations are mainly women [22].

Cardiovascular diseases are one of the leading causes of death, the most significant cause of hospitalizations, and the largest expense in the medical system [32,33]. Whether implementing telemedicine can reduce healthcare costs is still controversial [34,35]. Developed countries are increasingly facing the increasing burden of healthcare costs [36,37], which is increasingly reflected in times of crisis, such as the current COVID-19 pandemic, with the collapse of healthcare systems [38,39]. Similar to one Italian study [40], our study showed that the implementation of telemedicine could significantly reduce cost and burden in the medical system.

Finally, consumer and healthcare provider satisfaction also play a significant role in the treatment of the patient. Various studies have been conducted in this area, which have shown moderate satisfaction levels of both physicians and patients using telemedicine devices [40]. A study in Australia compared the usage of an ECG sensor with a standard Holter monitor [23]. Patients had to complete a questionnaire on the usefulness and ease of handling of the sensor. Of 47 patients, 21 previously had a Holter scan and responded that the sensor is more convenient (89.3%) and easier to use (90.5%). In our study, 82–98% of patients reported that the installation and use of an ECG sensor were easy, they were not bothered by the ECG sensor, and they gladly wore it and had no problems in its use. In similar studies, physicians were asked to complete a questionnaire about their satisfaction with this different telecardiology device [41,42]. The vast majority were extremely satisfied, and patient treatment was faster and more effective.

Our study was a prospective study, carried out in two health centers, and thus it captured the urban population as well as rural. It was carried out at the primary level, which gives it an even greater value, since no similar study had been carried out exclusively at the primary level until now. The questionnaires were also well-established. The study was observational, and patients of both groups were treated in concordance with medical guidelines and professional practice.

We also encountered some limitations in our study due to the fact that some family physicians were reluctant to participate and needed more reassurance to use the ECG sensors. Further limitations were the low number of male participants and the incomplete data for some physicians and their patients. Another issue was the lack of blinding of the physician choosing which intervention to follow.

## 5. Conclusions

The use of an ECG sensor confirms that the family physician’s diagnosis is correct and helps them distinguish between patients who need to be referred to a cardiologist and those who can be treated by primary healthcare providers. This study has proved that the use of the sensor is easy and useful for both the physician and patient because it can shorten the time taken to start treatment and reduce the cost of unnecessary referrals to cardiologists or hospital admittance.

## Figures and Tables

**Table 1:** Baseline characteristics of physicians involved.

Categorical Variables		n (%)
Sex	male	14 (47)
	female	16 (53)
Medical specialty	resident	1 (3)
	family medicine specialists	21 (70)
	without specialty	8 (26)
**Numerical variables**		**Mean ± SD**
Age (years)		41.87 ± 15.15
Years of service (years)		17.9 ± 11.17

**Table 2:** Baseline characteristics of patients involved by groups.

		Test Group	Control Group	Statistical Significance
Categorical Variables		n (%)	n (%)	
Gender	male	57 (28.5)	44 (22)	NS ^a^
	female	143 (71.5)	156 (78)	NS ^a^
Level of education	primary or high school	110 (55)	129 (65)	NS ^a^
	university, PhD	90 (45)	71 (35)	NS ^a^
Working status	working	94 (47)	92 (46)	NS ^a^
	unemployed or retired	106 (53)	108 (54)	NS ^a^
Lifestyle	smoking	41 (20.5)	92 (46)	*p* < 0.001 ^a^
	harmful alcohol consumption	130 (65)	120 (60)	NS ^a^
Associated diseases	hypertension	47 (27)	73 (36)	NS ^a^
	diabetes	28 (14)	26 (13)	NS ^a^
**Numerical variables**		**Mean ± SD**	**Mean ± SD**	
Age (years)		47.98 ± 17.37	51.61 ± 15.10	NS ^b^
Body mass index (kg/m^2^)		26.2 ± 5.26	25.2 ± 3.11	NS ^c^
Systolic blood pressure (mm Hg)		127.74 ± 14.94	140.12 ± 10.61	NS ^c^

^a^ χ^2^ test; ^b^ *t*-test for independent samples; ^c^ Mann–Whitney U-test; NS, not statistically significant (*p* > 0.05).

**Table 3:** Patient comparison by rhythm disorder suspicion.

		Anamnestic Suspicion n (%)	Clinical Suspicion n (%)	Both n (%)	Statistical Significance
Test group	all	91 (45)	68 (34)	41 (20)	*p* < 0.001
	female	68 (47)	45 (31)	30 (21)	NS ^a^
	male	23 (40)	23 (40)	11 (19)	
Control group	all	132 (66)	32 (16)	36 (18)	*p* < 0.001
	female	100 (64)	20 (12)	36 (23)	*p* < 0.001 ^a^
	male	32 (73)	12 (27)	0 (0)	

χ^2^ test; ^a^ χ^2^ test with adjusted standardized residuals; NS, not statistically significant (*p* > 0.05).

**Table 4:** Actions taken after receiving the electrocardiogram (ECG) sensor report for patients in the test and control groups.

		Rhythm Disorder Present	Treatment—Observation n (%)	Treatment—New Drug Administration n (%)	Referral to a Cardiologist n (%)
Test group	all	52 (26)	110 (55)	55 (27.5)	23 (11.5)
	female	36 (18)	78 (39)	37 (18.5)	16 (8)
	male	16 (8)	32 (16)	18 (9)	7 (3.5)
Control group	all	64 (32)	124 (62)	8 (4)	64 (32)
	female	40 (20)	108 (54)	0 (0)	44 (22)
	male	24 (12)	16 (8)	8 (4)	20 (10)
**Statistical significance**	all	NS	NS	*p* < 0.001	*p* < 0.001
	female	NS	NS ^a^	*p* < 0.001 ^a^	*p* = 0.009 ^a^
	male	NS	NS ^a^		

χ^2^ test; ^a^ Fisher exact; NS, not statistically significant (*p* > 0.05).
